# Food preferences and thyroid hormones in children and adolescents with obesity

**DOI:** 10.3389/fpsyt.2022.962949

**Published:** 2022-07-22

**Authors:** Daniela Staníková, Lea Krajčovičová, Linda Demková, Petronela Forišek-Paulová, Lucia Slobodová, Eva Vitariušová, Lubica Tichá, Barbara Ukropcová, Juraj Staník, Jozef Ukropec

**Affiliations:** ^1^Department of Pediatrics, Medical Faculty of Comenius University, National Institute for Children's Diseases, Bratislava, Slovakia; ^2^Department of Metabolic Disease Research, Biomedical Research Center, Institute of Experimental Endocrinology, Slovak Academy of Sciences, Bratislava, Slovakia; ^3^Medical Faculty of Comenius University, Institute of Pathophysiology, Bratislava, Slovakia

**Keywords:** food preference, children, obesity, thyroid hormones, FT4, high protein diet, high-fat diet, adolescents

## Abstract

**Background:**

Thyroid hormones profoundly affect energy metabolism but their interrelation with food preference, which might contribute to childhood obesity development, are much less understood. In this study, we investigated if thyroid hormone levels are associated with specific modulation of food preference and potentially linked to the level of obesity in children and adolescents.

**Methods:**

Interrelations between food preference and peripheral thyroid activity were examined in a population of 99 non-obese and 101 obese children and adolescents (12.8 ± 3.6 years of age, 111/89 F/M) randomly selected from the patients of the Obesity and Metabolic Disease Out-patient Research Unit at National Institute for Children's Diseases in Bratislava in a period between December 2017 and March 2020.

**Results:**

Children and adolescents with obesity had a lower preference for food rich in high sucrose and high-complex carbohydrates, while the preference for protein and fat-containing food and that for dietary fibers did not differ between obese and nonobese. In adolescents with obesity, free thyroxine (FT4) correlated positively with the preference for a high protein and high fat-rich diet, irrespective of the fatty acid unsaturation level. Moreover, FT4 correlated negatively with the preference for dietary fibers, which has been also exclusively found in obese adolescents. Individuals with obesity with higher FT4 levels had higher systemic levels of AST and ALT than the population with lower FT4. Multiple regression analysis with age, sex, BMI-SDS, and FT4 as covariates revealed that FT4 and male gender are the major predictors of variability in the preference for a diet high in protein, fat, and monounsaturated fatty acids. FT4 was the sole predictor of the preference for a diet containing saturated and polyunsaturated fatty acids as well as for a diet low in fiber.

**Conclusion:**

The link between free thyroxin levels and dietary preference for food rich in fat and protein is present exclusively in individuals with obesity. Higher serum FT4 was linked with elevated AST and ALT in children and adolescents with obesity, and FT4 was the best predictor for preference for food rich in fat and low in fiber. This may indicate that FT4 could contribute to the development of childhood obesity and its complications by modulating food preference.

## Introduction

Food preferences represent choices of most satisfying and favored food components based on an individual's sensory and energy needs, which could significantly influence eating behavior and contribute thus to the development of obesity. Food preferences are modulated by many different food-related and internal psychological and physiological factors and signals aimed at securing the proper food choices necessary for individual health and survival (1). Food preferences begin taking shape during fetal development and continue changing throughout life, influenced by biological, social, and environmental factors. Early childhood seems to be a critical period to establish food preferences persisting in later life (2). Parents play a crucial role in shaping food preferences (3). Their choices of what to serve to exert influence on their children's food preferences because children's familiarity with food may be as influential as any particular taste. The social and emotional context of food also influences preferences (4). Thus, food preferences result from interactions between learned behavior and genetic and environmental factors and could be modulated by hormonal signaling (5).

Hormonal modulation plays an important role in food choices as it may change the sense of taste and specifically modify food intake (5). Hormones known to directly or indirectly regulate food intake and perception include leptin, ghrelin, glucagon-like peptide 1 (GLP1), cholecystokinin, insulin, vasoactive intestinal peptide (VIP), or peptide YY (5, 6). Ghrelin and leptin play an important role in appetite control and respond promptly to changes in energy availability (fasting/refeeding), as besides the brain, both peptides have receptors in metabolically relevant peripheral tissues, and recent research shows that they are also present in olfactory mucosa (7). Leptin targets peripheral taste organs in lean but not db/db obese mice, by selectively suppressing gustatory neural and behavioral responses to sweet compounds without affecting responses to other taste stimuli (8). Synchronization of diurnal variation in leptin levels and sweet taste recognition thresholds provides indirect evidence that leptin also modulates sweet taste recognition in humans (5, 8). Leptin is an adipocyte-secreted hormone that regulates food intake and whole-body energy expenditure and modulates thus body weight in humans. Leptin also contributes to thyrotropin-releasing hormone regulation in the hypothalamus (9). However, obesity is associated with hyperleptinemia due to leptin resistance (10). Glucagon-like peptide 1 (GLP-1) is a hormone produced in the small intestine in response to food intake, endowed with the capacity to enhance satiety (11). GLP-1 was shown to be produced in two distinct subsets of mammalian taste cells, while the GLP-1 receptor is expressed on adjacent intragemmal afferent nerve fibers. Dramatically reduced taste responses to sweet taste were found in *glp1-/-* mice, indicating that GLP-1 signaling might be critical for the maintenance of sweet taste sensitivity (12) and has a potential role in signaling sour or umami taste (13). Moreover, cholecystokinin (CCK) and peptide YY seem to have a role in processing perception of bitter compounds (14, 15). The above-mentioned capacity of hormones to modulate taste perception provides clear evidence for their involvement in food preference and macronutrient intake (5).

Hormones could also have an important role in the hedonic mechanisms of food intake influencing the dopaminergic system. Similarly to addictive drugs, palatable foods, which are rich in fat and sugar content, can significantly activate the dopamine reward system (16). Based on the role of the dopamine reward system in food-seeking behavior, considerable evidence has revealed that there is an interplay between the homeostatic regulator and dopamine system, such that homeostatic regulators of food intake interact with the dopamine reward system to exert an inhibitory or enhancing effect on food intake (17). It has been shown that leptin and insulin inhibit dopamine neurons, while ghrelin activates them. Hommel et al. established that ventral tegmental area dopamine neurons express the leptin receptor, and in response to leptin, these ventral tegmental area leptin receptors are activated and suppress the activity of dopamine neurons (18). Administration of leptin to the ventral tegmental area was found to decrease food consumption, while knockdown of leptin receptors in the ventral tegmental area resulted in an increase in food intake, locomotor activity, and hedonic feeding (17).

Thyroid hormones are important determinants of energy expenditure and appetite regulation as they are involved in the regulation of resting metabolic rate*, de novo* gluconeogenesis, and liver and adipose tissue lipolysis and lipogenesis (19). It is plausible to think that the regulation of these metabolic processes might be tightly related to the regulation of taste perception and food preferences. To date, only a few studies in adults with hypothyroidism provided conflicting results on the effects of thyroid hormone replacement therapy on olfaction, taste, and food preferences (20, 21). Thyroid hormone secretion in children and adolescents is regulated similarly to that in adults, mainly through the hypothalamic-pituitary-thyroid axis (22). However, thyroid hormones in children and adolescents additionally have multiple functions affecting growth and development, including the development of the brain (23). Therefore, dysregulation of thyroid hormone secretion at young age can have serious developmental consequences.

Obesity is a serious condition, and already childhood obesity could have some serious consequences in later life. It was shown that almost 90% of the children who were obese at 3 years of age were overweight or obese in adolescence (24), around 80% of obese adolescents will still be obese in adulthood, and around 70% will be obese over the age of 30 years (25). Obesity is often associated with altered thyroid hormone levels, and their change could predict change in body weight. Among nonobese individuals in the Pizarra study (*n* = 937), those with higher levels of free triiodothyronine (FT3) or free thyroxine (FT4) had 3 times higher risk of becoming obese during the 6-year follow-up as compared with their counterparts with low thyroid hormone levels. Moreover, thyrotropin (TSH) and FT4 correlated with leptin, and FT3 correlated negatively with adiponectin (26, 27). A recent study has shown that obesity is associated with altered gene expression in human taste buds (28). It is widely accepted that metabolic surgery affecting the digestive as well as the hormonal milieu of the human body clearly modulates food preference, which might be linked with taste and odor sensing (29, 30). In this study, we hypothesized that thyroid hormones could be related to the modulation of food preferences and contribute thus to childhood obesity. Therefore, we investigated if thyroid hormone levels across different age groups are paralleled by specific modulation of food preference and, thus, could be potentially linked to the level of obesity in children and adolescents.

## Materials and methods

### Study population

In this study, we examined interrelations between food preference and peripheral thyroid activity in a population of 99 non-obese and 101 obese adolescents selected from the patients of obesity and metabolic disease out-patient research unit at the National Institute for Children's Diseases in Bratislava examined between December 2017 and March 2020. All children and adolescents with obesity, thyroid disorder, or healthy children aged >4 years referred to a pediatric endocrinologist with suspicion of thyroid disease were included. Individuals with any other chronic or acute metabolic disorder including diabetes mellitus and individuals with genetic syndromes were excluded. A total of 11 participants (5.2%) with incomplete food preference questionnaires were also excluded. There was no additional dropout. Participants did not have any specific medical-based dietary recommendations. At the time of the examination, anthropometric data were recorded, and blood was sampled for biochemical analyses while children and adolescents with or without the assistance of their parents/guardians filled out the food preference questionnaire.

### Anthropometry

Anthropometric measurements were taken by trained nurses according to standardized protocols. Body mass index (BMI) was calculated as weight divided by the square of body height. The standard deviation score (SDS) for BMI was calculated using local reference values (31). Categories for the BMI SDS score were defined as follows: non-obese children and adolescents with a BMI SDS <1.88 and obese with BMI SDS ≥ 1.88. Both non-obese and obese patients were further stratified to low and high FT4 subpopulations according to the median level of free thyroxine.

### Assessing food preferences

Food preferences were assessed using the validated food preference questionnaire (32). The food preference questionnaire requires patients to rate 72 food items on a 9-point scale ranging from “dislike a lot” (1), neutral feelings about the food (5) to “like a lot” (9). If patients did not have a memory of trying the particular food item or if they have never tested it, “I don't know” was selected. Food items were classified into 12 groups according to the nutrient composition; eight of them were used in this study (high sugar score, high complex carbohydrate score, high protein score, high-fat score, high saturated fatty acid score, high monounsaturated fatty acid score, high polyunsaturated fatty acid score, and low dietary fiber score).

### Biochemical analyses

Blood for biochemical and hormonal analyses was collected from the serum tubes between 7.30 and 10.00 a.m. The samples were processed and analyzed as routine fresh samples by the clinical service laboratory at the National Institute for Children's Diseases. Thyroid hormone levels and biochemical markers describing the metabolic health in obese individuals were selected for the analyses.

### Statistics

Variables were checked for normality using the Shapiro–Wilk test. Normally distributed data are expressed as the mean ± SD. Non-normally distributed data (aspartate aminotransferase (AST), alanine aminotransferase (ALT), and ALT/AST ratio) are presented as the median and interquartile range. Differences between the two groups were tested using the two-sided Student's *t*-test or with Mann–Whitney *U* test and by Fisher's test for binary data. Univariate associations of food preferences with the other clinical variables were calculated using Pearson's correlation and linear regression. Multivariate associations between selected variables were determined using forward linear multiple regression analyses. Food preferences were used as dependent variables, and age sex, BMI-SDS, and FT4 were used as covariates. *p* < 0.05 was considered statistically significant. Statistical analyses were performed using the SPSS version 27 (IBM, USA), JMP (USA), and GraphPad Prism 7 (GraphPad, USA) software.

## Results

### Study population

This study included 200 children and adolescents; 99 were non-obese, and 101 had obesity. Characteristics of the study population are presented in Table 1.

**Table 1:** Basic characteristics of the study population.

	**All**	**Non-obese**	**Obese**	**p**
Age (years)	12.95 ± 3.29 (200)	12.48 ± 3.25 (99)	13.41 ± 3.28 (101)	0.047
Sex (% of girls)	55.5 (200)	67.7 (99)	43.6 (101)	<0.001
Height (cm)	158.14 ± 17.74 (200)	154.29 ± 18.25 (99)	161.91 ± 16.47 (101)	0.002
Height SDS	2.16 ± 2.68 (200)	0.07 ± 1.41 (99)	0.42 ± 1.17 (101)	0.052
Body weight (kg)	65.1 ± 29.11 (200)	47.56 ± 15.91 (99)	82.29 ± 28.9 (101)	<0.001
BMI (kg/m^2^)	24.95 ± 7.85 (200)	19.3 ± 3.19 (99)	30.48 ± 7.08 (101)	<0.001
BMI SDS	0.25 ± 1.3 (200)	0.16 ± 0.99 (99)	4.12 ± 2.35 (101)	<0.001
TSH (mU/l)	3.01 ± 1.83 (200)	2.72 ± 1.53 (99)	3.31 ± 2.06 (101)	0.023
FT4 (pmol/l)	15.53 ± 2.6 (200)	16.03 ± 2.88 (99)	15.04 ± 2.21 (101)	0.007
FT3 (pmol/l)	6.96 ± 1.1 (142)	6.94 ± 1.11 (68)	6.97 ± 1.09 (74)	0.835
High sugar score	5.69 ± 1.36 (200)	5.99 ± 1.33 (99)	5.4 ± 1.35 (101)	0.002
High complex carbohydrate score	5.85 ± 1.25 (200)	6.04 ± 1.23 (99)	5.68 ± 1.24 (101)	0.041
High protein score	5.54 ± 1.52 (200)	5.57 ± 1.54 (99)	5.52 ± 1.51 (101)	0.820
High fat score	5.71 ± 1.38 (200)	5.86 ± 1.34 (99)	5.56 ± 1.41 (101)	0.122
Low fiber score	5.94 ± 1.38 (133)	6.11 ± 1.23 (69)	5.75 ± 1.51 (64)	0.131
Foods cotaining ≥1.5 g of saturated FA per 100 g	5.71 ± 1.32 (133)	5.93 ± 1.22 (69)	5.47 ± 1.38 (64)	0.041
Foods cotaining ≥1.5 g of polyunsaturated FA per 100 g	5.76 ± 1.3 (133)	5.97 ± 1.22 (69)	5.54 ± 1.35 (64)	0.057
Foods cotaining ≥1.5 g of monounsaturated FA per 100 g	5.73 ± 1.28 (133)	5.95 ± 1.21 (69)	5.49 ± 1.33 (64)	0.040

*Data are expressed as the mean ± standard deviation. TSH, thyroid-stimulating hormone; FT4, free thyroxine; FT3, free triiodothyronine; BMI, body mass index; SDS, standard deviation score; FA, fatty acids. The p-value for the difference between populations of lean and obese children and adolescents, value in parentheses (number of participants). All significant values (p <0.005) are in bold*

### Food preference in nonobese and obese children and adolescents

**Figure 1:**
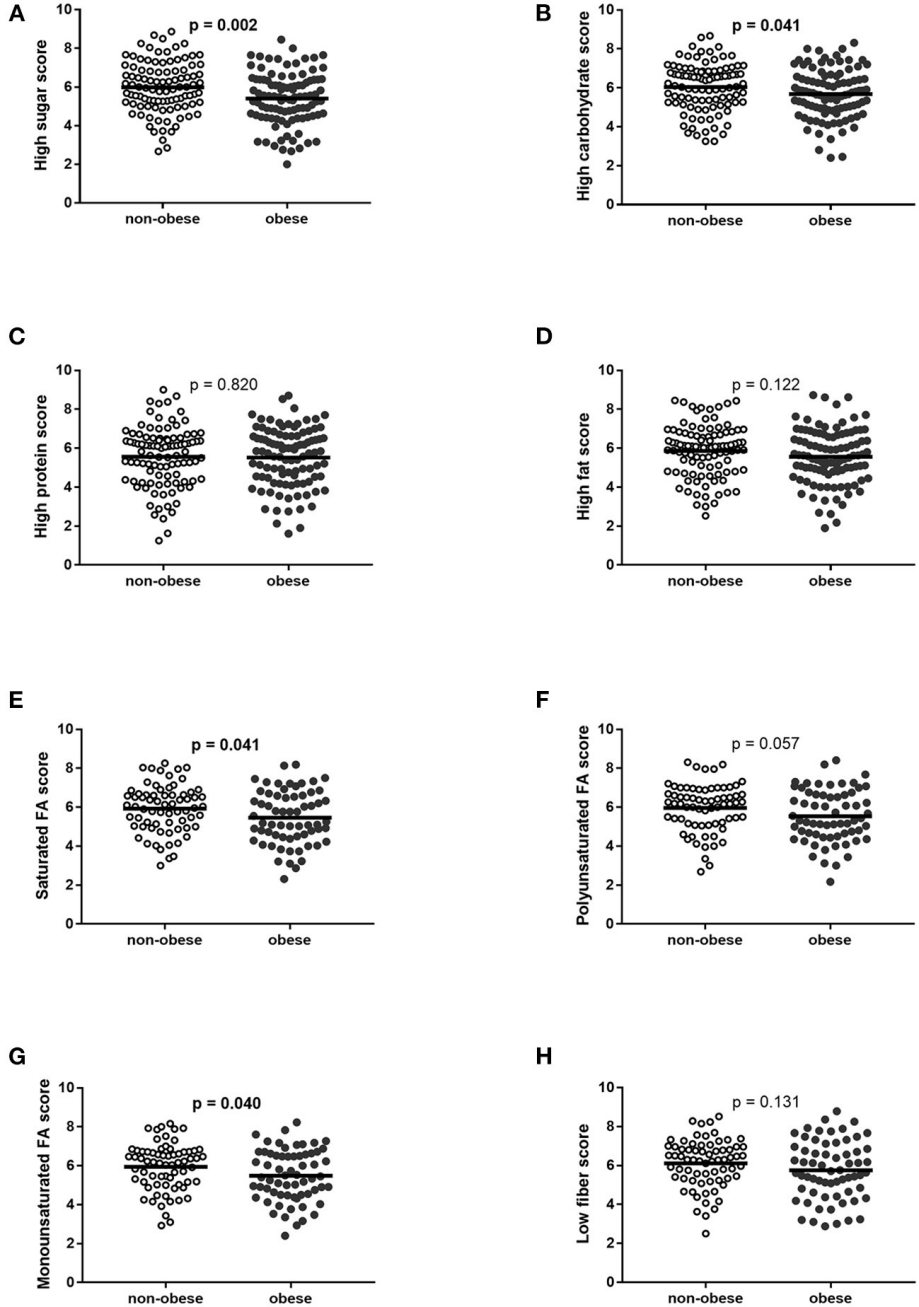
Food preferences in non-obese and obese children and adolescents. **(A)** High sugar score, **(B)** high carbohydrate score, **(C)** high protein score, **(D)** high-fat score, **(E)** high saturated fatty acid score, **(F)** high polyunsaturated fatty acid score, **(G)** high monounsaturated fatty acid score, and **(H)** low fiber score. Displayed as mean and confidential intervals for the mean. Differences were calculated using the *t-*test. FA, fatty acids.

Obese participants (57 boys and 44 girls) indicated a lower preference for food rich in high sucrose and high complex carbohydrate, while the preference for protein and fat-containing food and for dietary fibers was not different in obese and non-obese groups. Participants with obesity had also a lower preference for food rich in high saturated and monounsaturated fatty acids, and the trend was found for a lower preference to eat a diet rich in polyunsaturated fatty acids (Table 1, Figure 1).

### Thyroid hormones and food preferences

Interrelations between food preference parameters and serum levels of FT4, FT3, and TSH were examined in both, obese and non-obese children and adolescent populations. Children and adolescents with obesity had lower serum FT4 levels compared with non-obese participants (Table 1).

**Figure 2:**
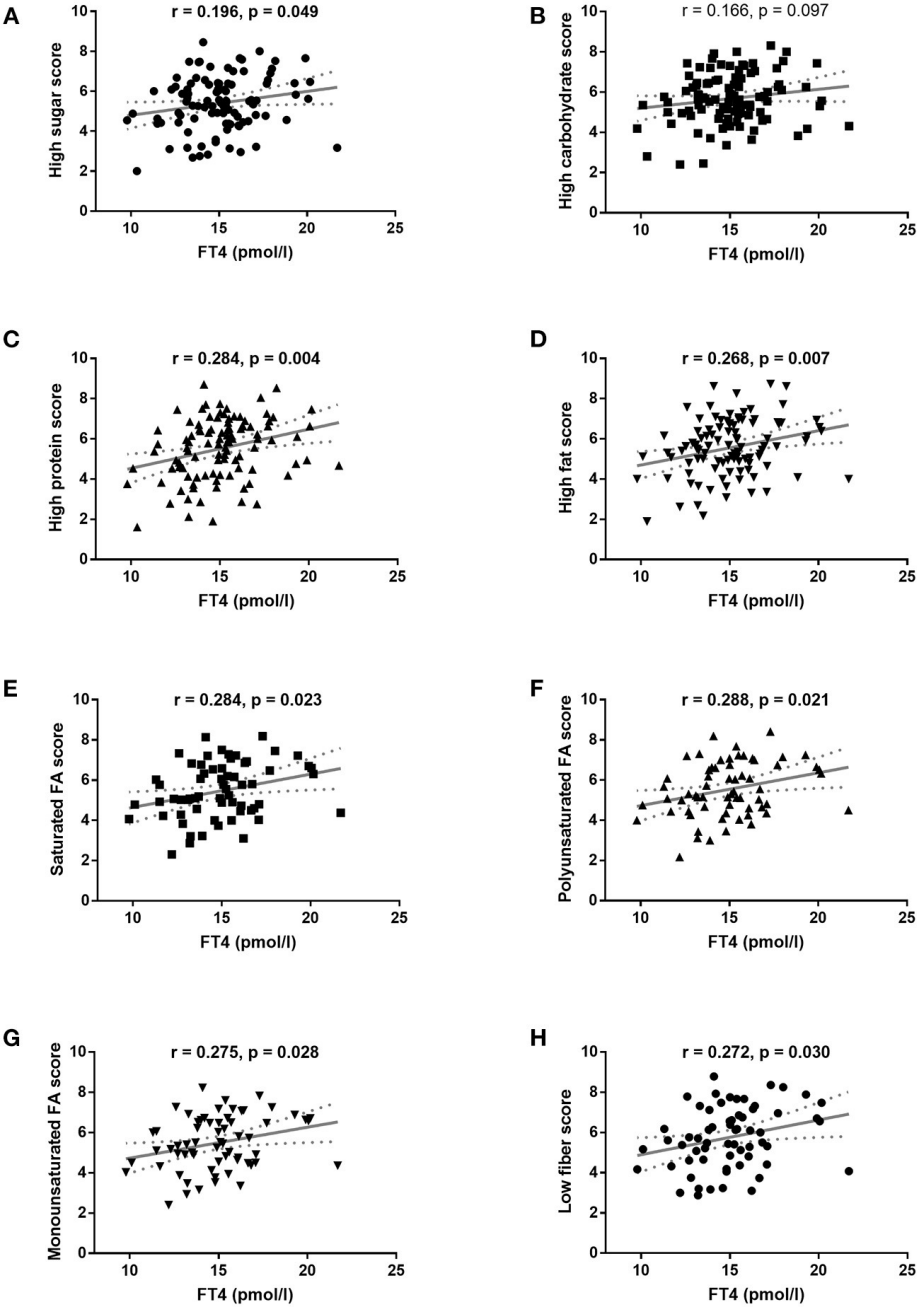
Pearson's correlation of food preferences with FT4 serum levels in obese children and adolescents. **(A)** High sugar score, **(B)** high carbohydrate score, **(C)** high protein score, **(D)** high-fat score, **(E)** high saturated fatty acid score, **(F)** high polyunsaturated fatty acid score, **(G)** high monounsaturated fatty acid score, and **(H)** low fiber score. Associations were calculated with Pearson's correlation. The regression line with 95% confidence intervals was calculated in linear regression analysis.

Serum FT4 levels in children and adolescents with obesity correlated positively with their preference for food containing high protein, high-fat diet, as well as with preference for a diet rich in saturated, monounsaturated, and polyunsaturated fatty acids, and with a diet low in dietary fibers (Figure 2). It is important to note that no significant correlations were found between FT4 and food preferences in the non-obese population of children and adolescents (data not shown).

Serum FT3 levels in the obese group correlated positively with dietary preference for food rich in carbohydrates (*r* = 0.239, *p* = 0.040), saturated (*r* = 0.356, *p* = 0.019), monounsaturated (*r* = 0.322, *p* = 0.035), and polyunsaturated fatty acids (*r* = 0.345, *p* = 0.024), as well as for food with low fiber content (*r* = 0.336, *p* = 0.028). In the non-obese population, FT3 levels correlated negatively with the preference for high carbohydrate food (*r* = −0.307, *p* = 0.011).

Serum levels of TSH did not correlate with any of the examined food preferences in neither obese nor non-obese populations.

### Stratification of the patient population by the levels of FT4 reveals distinct patients' characteristics

Stratification of the patient population into low FT4 and high FT4 subgroups revealed no additional FT4-related regulation of the dietary preference for food rich in simple and complex carbohydrates. However, participants with obesity and high FT4 levels showed a higher preference for food high in protein and fat. More detailed analysis revealed that higher FT4 levels were linked to an increased dietary preference for food items containing more than 1.5 g of fat per 100 g serving. This held true for food containing saturated, monounsaturated, and polyunsaturated fatty acids. Moreover, children and adolescents with obesity who had high FT4 levels preferred a diet with low fiber contents (Table 2).

**Table 2:** Selected phenotypes in children and adolescents stratified according to FT4 serum levels.

	**Non-obese**			**Obese**		
	**Low FT4**	**High FT4**	**p**	**Low FT4**	**High FT4**	**p**
Age (years)	12.19 ± 2.81 (50)	12.77 ± 3.65 (49)	0.378	13.54 ± 3.28 (49)	13.27 ± 3.31 (52)	0.680
Sex (% of girls)	70.0 (50)	65.3 (49)	0.671	51.0 (49)	36.5 (52)	0.164
BMI SDS	0.26 ± 1.03 (50)	0.07 ± 0.95 (49)	0.341	4.11 ± 1.99 (49)	4.12 ± 2.66 (52)	0.98
TSH (mU/l)	2.87 ± 1.64 (50)	2.56 ± 1.4 (49)	0.306	3.58 ± 2.33 (49)	3.05 ± 1.75 (52)	0.198
FT4 (pmol/l)	13.83 ± 1.56 (50)	18.28 ± 2.05 (49)	<0.001	13.33 ± 1.28 (49)	16.66 ± 1.58 (52)	<0.001
FT3 (pmol/l)	7.13 ± 1.01 (34)	6.74 ± 1.19 (34)	0.144	7.02 ± 0.94 (34)	6.93 ± 1.22 (40)	0.728
Fasting serum glucose (mmol/l)	4.89 ± 0.35 (27)	4.86 ± 0.45 (20)	0.748	4.88 ± 0.39 (32)	4.81 ± 0.42 (30)	0.494
Urea (mmol/l)	3.71 ± 1 (13)	3.98 ± 1.27 (9)	0.582	3.81 ± 0.84 (22)	4.15 ± 0.83 (25)	0.166
Creatinine (mmol/l)	48.53 ± 9.19 (30)	52.81 ± 10.79 (26)	0.157	51.08 ± 12.98 (36)	53.76 ± 14.32 (33)	0.419
Uric acid (μmol/l)	249.19 ± 68.94 (26)	261.62 ± 69.06 (21)	0.542	312.65 ± 78.3 (34)	325.21 ± 69.4 (34)	0.486
AST (μkat/l)	0.39 (0.32–0.45) (30)	0.36 (0.31–0.47) (27)	0.614*	0.39 (0.35–0.44) (35)	0.46 (0.37–0.53) (33)	0.013*
ALT (μkat/l)	0.24 (0.21–0.35) (31)	0.25 (0.22–0.31) (27)	0.857*	0.35 (0.27–0.45) (35)	0.44 (0.37–0.77) (33)	0.006*
ALT/AST ratio	0.866 (0.56–0.86) (30)	0.68 (0.58–0.81) (27)	0.955*	0.89 (0.74–1.19) (35)	1.07 (0.82–1.65) (33)	0.111*
GGT (μkat/l)	0.24 ± 0.13 (9)	0.17 ± 0.03 (4)	0.313	0.33 ± 0.18 (17)	0.38 ± 0.24 (21)	0.492
ALP (μkat/l)	3.75 ± 1.2 (14)	2.95 ± 1.52 (10)	0.162	3.9 ± 1.84 (19)	3.58 ± 1.5 (24)	0.532
Total-cholesterol (mmol/l)	3.88 ± 0.6 (28)	4.34 ± 0.94 (24)	0.037	4.15 ± 0.53 (34)	4.25 ± 0.71 (32)	0.493
HDL-cholesterol (mmol/l)	1.38 ± 0.25 (16)	1.52 ± 0.35 (14)	0.206	1.24 ± 0.27 (34)	1.27 ± 0.28 (30)	0.688
LDL-cholesterol (mmol/l)	2.36 ± 0.59 (15)	2.73 ± 0.6 (14)	0.104	2.62 ± 0.53 (34)	2.71 ± 0.71 (30)	0.582
Triglycerides (mmol/l	0.97 ± 0.5 (24)	0.9 ± 0.45 (23)	0.596	1.2 ± 0.62 (34)	1.09 ± 0.46 (32)	0.400
Insulin (mU/l)	14.78 ± 9.3 (10)	9.36 ± 3.17 (7)	0.162	22.81 ± 15.06 (30)	16.41 ± 9.72 (28)	0.062
High sugar score	5.98 ± 1.3 (50)	6 ± 1.36 (49)	0.939	5.25 ± 1.43 (49)	5.55 ± 1.25 (52)	0.259
High complex carbohydrate score	5.98 ± 1.29 (50)	6.1 ± 1.18 (49)	0.637	5.47 ± 1.28 (49)	5.88 ± 1.19 (52)	0.098
High protein score	5.64 ± 1.62 (50)	5.49 ± 1.47 (49)	0.64	5.08 ± 1.6 (49)	5.93 ± 1.3 (52)	0.004
High fat score	5.93 ± 1.42 (50)	5.8 ± 1.27 (49)	0.636	5.26 ± 1.47 (49)	5.85 ± 1.3 (52)	0.033
Low fiber score	6.22 ± 1.29 (35)	6 ± 1.18 (34)	0.462	5.35 ± 1.55 (32)	6.15 ± 1.37 (32)	0.034
Score for foods cotaining ≥1.5g of saturated FA per 100g	6.06 ± 1.28 (35)	5.8 ± 1.17 (34)	0.395	5.08 ± 1.4 (32)	5.85 ± 1.26 (32)	0.023
Score for foods cotaining ≥1.5g of polyunsaturated FA per 100g	6.09 ± 1.27 (35)	5.84 ± 1.17 (34)	0.402	5.17 ± 1.39 (32)	5.9 ± 1.22 (32)	0.029
Score for foods cotaining ≥1.5g of monounsaturated FA per 100g	6.07 ± 1.24 (35)	5.82 ± 1.18 (34)	0.409	5.15 ± 1.37 (32)	5.83 ± 1.21 (32)	0.039

Normally distributed data are expressed as the mean ± standard deviation. Non-normally distributed data* (AST, ALT, and ALT/AST ratio) are presented as the median and interquartile range. TSH, thyroid-stimulating hormone; FT4, free thyroxine; FT3, free triiodothyronine; BMI, body mass index; SDS, standard deviation score; AST, aspartate aminotransferase; ALT, alanine aminotransferase; GGT, gamma-glutamyl transferase; ALP, alkaline phosphatase; FA, fatty acids. Value in parentheses (number of participants). All significant values (p <0.005) are in bold.

Children and adolescents with obesity and high circulating FT4 were characterized by higher AST and ALT serum levels as compared with their counterparts with low FT4 levels. No significant differences in obesity level (BMI-SDS), fasting glycemia, urea, creatinine, uric acid, gamma-glutamyl transferase, alkaline phosphatase, and serum lipids were found in obese children and adolescents in association with low or high levels of FT4 (Table 2).

It is important to note that no significant differences in food preference were found in non-obese children and adolescents in relation to low and/or high FT4 serum levels.

### The best predictors of dietary preference

Multiple regression analysis aimed at identifying the best predictors of variability in dietary preference in children and adolescents with obesity encompassed age, sex, BMI-SDS, and FT4 as covariates. The results revealed that FT4 and sex are the major predictors of variability in the preference for a diet high in protein, fat in general, and monounsaturated fatty acids in particular. High levels of FT4 were the sole predictor of the higher preference for a diet containing saturated and polyunsaturated fat as well as for a diet low in fiber (Table 3).

**Table 3:** Multiple regression analysis of food preferences in obese children and adolescents.

**Model summary**	**Dependent**	**Independent**	**ΔR^2^**	**β ±SEM**	***p*** **-Value**
R^2^ = 0.186; *p* < 0.001; *n* = 101	High protein score	Sex	0.105	0.32 ± 0.10	0.001
		FT4	0.081	0.28 ± 0.10	0.002
R^2^ = 0.180; *p* < 0.001; *n* = 101	High fat score	Age	0.087	−0.27 ± 0.12	0.005
		FT4	0.048	0.22 ± 0.11	0.020
		Sex	0.045	0.21 ± 0.11	0.023
R^2^ = 0.074; *p* = 0.030; *n* = 64	Low fiber	FT4	0.074	0.23 ± 0.12	0.030
R^2^ = 0.081; *p* = 0.023; *n* = 64	Foods containing ≥1.5g of saturated FA per 100g	FT4	0.081	0.25 ± 0.12	0.023
R^2^ = 0.083; *p* = 0.021; *n* = 64	Foods containing ≥1.5g of polyunsaturated FA per 100g	FT4	0.083	0.26 ± 0.12	0.021
R^2^ = 0.135; *p* = 0.012; *n* = 64	Foods containing ≥1.5g of monounsaturated FA per 100g	FT4	0.075	0.25 ± 0.12	0.024
		Sex	0.059	0.24 ± 0.12	0.045

*Analyzed in forward linear multiple regression analyses. Covariates: age, sex, BMI-SDS, and FT4. FT4, free thyroxine; BMI, body mass index; SDS, standard deviation score; FA, fatty acids. All significant values (p <0.005) are in bold*.

## Discussion

This study clearly shows that higher levels of FT4 are associated with a higher preference for protein and a fat-rich diet, regardless of the level of fatty acid unsaturation, and with a lower preference for dietary fibers in children and adolescents with obesity. Children and adolescents with obesity and higher FT4 levels (stratified by median value) had higher systemic levels of AST and ALT. Multiple regression analysis revealed that higher FT4 is an independent predictor of preference to consume a diet low in fiber and high in protein and fat content and irrespective of the fatty acid unsaturation level among children and adolescents with obesity. No significant associations between thyroid hormones and food preference scores were found in non-obese children and adolescents.

To the best of our knowledge, this is the first study bringing evidence indicating that the thyroid hormones might modulate food preference in children and adolescents with obesity. Several studies have already pointed out links between thyroid hormones and dietary fat intake. Kalicanin et al. used a food frequency questionnaire to examine dietary habits in 491 patients with Hashimoto's thyroiditis (HT) and 433 controls. They found that the consumption of the plant oils correlated positively with triiodothyronine levels in the entire cohort of HT patients, as well as in those on LT4 therapy (33). Matana et al. used logistic regression analysis to evaluate associations between dietary factors and plasma thyroid peroxidase antibodies (TPO-Ab) and/or thyroglobulin antibodies (Tg-Ab) in 462 TPO-Ab and/or Tg-Ab positive antibodies and 1,425 were negative individuals. A food frequency questionnaire was used to evaluate dietary habits, and logistic regression analysis showed that the consumption of animal fat and butter was associated with the presence of plasma antithyroid peroxidase (TPO-Ab) and/or thyroglobulin (Tg-Ab) antibodies (34). The fact that the high-fat diet induces ectopic lipid deposition in the thyroid gland of young adult Sprague-Dawley rats, resulting in decreased thyroid function and manifested by a decline of thyroxine, FT4, and increased thyrotropin allows us to speculate that lower levels of FT4 in some children and adolescents with obesity might indicate obesity-related impairment of thyroid function and counter-regulatory inhibition of dietary fat intake (35, 36). The link between thyroid hormones and food preference/taste perception has also been documented in a study examining interrelations between type 2 taste receptors (TAS2R), activated by bitter-tasting compounds with thyroid function. TAS2Rs were shown to play an important role in the modulation of thyroid hormone (T3/T4) production (37).

The fact that the associations of thyroid hormones and food preferences were, in our study, exclusive to children and adolescents with obesity supports the hypothesis that obesity alters the regulation of thyroid hormones and modulates thus the food preferences in order to limit the intake of high fat-containing diet. A recent Australian study provided clear evidence of the mutual interrelationship between obesity and taste perception. Authors have shown specific obesity-related differences in the gene expression profile of human fungiform papillae. Very distinct was also the taste bud cellular microenvironment, which could alter taste bud function and therefore modulate taste perception and food intake (28). The alterations of thyroid hormone levels in common obesity are believed to be rather a consequence than a cause of obesity (26), and we explored the hypothesis that the modulation of food preference could also be a part of the regulatory circuit. It is also known that thyroid hormones modulate resting energy expenditure and might therefore be involved in modulating energy allostasis and body weight control (38). However, the pathophysiological mechanisms by which thyroid hormones regulate the balance between energy intake and expenditure are not fully explained, and one of the possibilities could be *via* modulating food preferences. We could also speculate that the positive association of serum FT4 levels with food preferences in obese children and adolescents might be one of the reasons why patients with obesity and subclinical hypothyroidism mostly do not lose weight on the L-thyroxin treatment. Higher serum levels of FT4 caused by substitution therapy should increase energy expenditure. However, this effect could be balanced with the increased preferences for a diet rich in fat and protein while containing only a limited amount of dietary fiber. Therefore, our results support recommendations that thyroxine treatment should be carefully considered in children and adolescents with obesity. In people who do require thyroxine replacement therapy, it would be beneficial to use not only serum TSH but also FT4 concentrations to monitor the treatment. A further potential application of our results in clinical practice may also be in the identification of individuals with obesity and higher FT4 concentrations that may be at risk for the development of obesity-related hepatopathy. The clinical utility of our results could be further supported by the prospective follow-up of this patient population as well as by including patients with abnormal or extreme levels of thyroid hormones.

### Limitations

Several factors that were not evaluated in this study, including social and cultural influences, could have an impact on food preferences. There was also a significant gender imbalance in the non-obese and obese participants which could influence the comparisons between these two groups. Moreover, associations between thyroid hormones and food preferences in obese individuals were found in a specific age group of children and adolescents. Therefore, further studies in other age groups will be needed to generalize our presumptions.

## Conclusion

Food preference could be an important predictor of weight changes and certainly should be considered in the management of childhood obesity. Association with serum levels of thyroid hormones provides an interesting link, with the potential clinical utility of FT4 concentrations in monitoring thyroxine replacement therapy in people with obesity or in identifying persons at higher risk of developing specific complications of obesity.

It is important to note that the lower food preference for protein and fat that we observed in children and adolescents with obesity and low FT4 levels could provide specific protection from obesity-related lipotoxicity as evidenced by lower AST and ALT. Collectively, it is possible to speculate that lower FT4 might signal the need for protection against metabolic disease in children and adolescents with obesity, which might be exemplified by a lower preference for protein and fat-rich food.

## Data availability statement

The raw data supporting the conclusions of this article will be made available by the authors, without undue reservation.

## Ethics statement

The studies involving human participants were reviewed and approved by Ethics Committee of the National Institute for Children's Diseases in Bratislava. Written informed consent to participate in this study was provided by the participants' legal guardian/next of kin.

## Author contributions

DS, BU, JU, and JS: original idea development. DS, LD, PF-P, LS, EV, LT, and JS: data collection. DS, JU, and JS: analysis. DS and LK: manuscript preparation. JU, BU, and JS: critical reading and editing of the manuscript. All authors contributed to the study design, reviewed the manuscript critically, and approved the final version.

## Funding

This study was supported by research grants VEGA 1/0308/19, KEGA 053UK-4/2020, European Regional Development Fund–IMTS313011V344 (BU), and COST CA19101 (BU).

## Conflict of interest

The authors declare that the research was conducted in the absence of any commercial or financial relationships that could be construed as a potential conflict of interest.

## Publisher's note

All claims expressed in this article are solely those of the authors and do not necessarily represent those of their affiliated organizations, or those of the publisher, the editors and the reviewers. Any product that may be evaluated in this article, or claim that may be made by its manufacturer, is not guaranteed or endorsed by the publisher.
